# Survival of older patients after critical care

**DOI:** 10.1186/cc12414

**Published:** 2013-03-19

**Authors:** D Ashton-Cleary, R Sinclair

**Affiliations:** 1 Royal Cornwall Hospital, Truro, UK

## Introduction

We aimed to assess the survival of older patients after admission to critical care compared with the population in general.

## Methods

We reviewed admission data for a 12-bed critical care unit in a district general hospital in the UK between 2001 and 2010. We calculated critical care and hospital discharge survival rates and studied the trend in these through the decade. We searched hospital records to establish whether these patients were still alive at November 2012 to determine life-expectancy (minimum 22-month follow-up on all patients). This was compared with UK Office of National Statistics data for the local population [1]. Discharge destination (care facility or home address) was recorded as a surrogate marker of change in quality of life post discharge.

## Results

A total of 481 patients aged 80 or over were admitted between 1 January 2001 and 31 December 2010, representing 9.6% of the admissions during this time with minimal annual change. Admission age ranged from 80 to 96 years (median 83) with a median APACHE II score of 20 (6 to 48, IQR 16 to 25). In total, 318 (66.1%) survived to critical care discharge and 256 (53.2%) survived to hospital discharge; 228 (89.0%) went home rather than to a care facility. Those under 80 years demonstrated 80.6% critical care survival, 65.5% hospital survival and a median APACHE II score of 17 (0 to 51, IQR 13 to 23). Eighty patients died within the 22-month follow-up period with an average life expectancy of 7.4 months. None reached life expectancy (average curtailment of 5.6 years compared with age-matched and sex-matched population statistics). Neither of these figures changed significantly through the decade. However, 10-year follow-up on 2001/2002 admissions showed that, despite this early mortality effect, the average post-discharge lifespan was 5.1 years with 2.2 years shortfall in life expectancy.

## Conclusion

Older people represent a growing proportion of the population although their representation in the critical care population remained constant in this 10-year study. These patients had a slightly higher median APACHE II score and 14.5% greater critical care mortality than the younger patients. The majority of survivors were able to go home; however, 31% died within 22 months with significant life expectancy curtailment, surviving on average only 7.4 months after discharge; this has not changed in the last 10 years. Those who survived this initial period (69%) had a much better outlook. This information may be vital to patients and physicians when discussing admission to critical care.
